# Atomic Scale Formation Mechanism of Edge Dislocation Relieving Lattice Strain in a GeSi overlayer on Si(001)

**DOI:** 10.1038/s41598-017-12009-y

**Published:** 2017-09-20

**Authors:** E. Maras, L. Pizzagalli, T. Ala-Nissila, H. Jónsson

**Affiliations:** 1 COMP Center of Excellence Aalto University School of Science, FI-00076 Aalto, Espoo Finland; 2 Department of Applied Physics, Aalto University School of Science, FI-00076 Aalto, Espoo Finland; 3 Department of Physics and Mechanics of Materials, Institut Pprime, CNRS UPR 3346, Université de Poitiers, SP2MI, BP30179, 86962 Futuroscope Chasseneuil, France; 4 Department of Physics, Box 1843, Brown University, Providence, RI 02912-1843 USA; 5 Departments of Mathematical Sciences and Physics, Loughborough University, Loughborough, Leicestershire, LE11 3TU United Kingdom; 6 Faculty of Physical Sciences, University of Iceland, 107 Reykjavík, Iceland

## Abstract

Understanding how edge misfit dislocations (MDs) form in a GeSi/Si(001) film has been a long standing issue. The challenge is to find a mechanism accounting for the presence of these dislocations at the interface since they are not mobile and cannot nucleate at the surface and glide towards the interface. Furthermore, experiments can hardly detect the nucleation and early stages of growth because of the short time scale involved. Here we present the first semi-quantitative atomistic calculation of the formation of edge dislocations in such films. We use a global optimization method and density functional theory calculations, combined with computations using potential energy functions to identify the best mechanisms. We show that those previously suggested are relevant only for a low film strain and we propose a new mechanism which accounts for the formation of edge dislocations at high film strain. In this one, a 60° MD nucleates as a “split” half-loop with two branches gliding on different planes. One branch belongs to the glide plane of a complementary 60° MD and therefore strongly favors the formation of the complementary MD which is immediately combined with the first MD to form an edge MD.

## Introduction

The formation of a stable and well ordered overlayer on a substrate even when there is a large lattice mismatch is of importance in many applications. Thin films of GeSi deposited on a Si(001) substrate are among the most studied heteroepitaxial systems and have several applications in optoelectronics^1,2^, photovoltaics^3^ and microelectronics^4^. The main experimental challenge is to obtain a high quality film with a very low content of dislocations. However, the large difference (about 4%) in lattice constant between Ge and Si induces a large misfit strain in the film. For deposition at high temperatures the strain is partially released by growth of islands in the Stranski-Krastanow mode^5^ and no clean film can be obtained. For deposition at low temperatures, continuous films can be realized and the strain is released by plastic relaxation (i.e. through nucleation, glide and interaction of dislocations). In low to moderate Ge content GeSi/Si(001) films, plastic relaxation has been characterized from TEM experiments^6^. It occurs mainly through the nucleation from the surface of half-loop dislocations with Burgers vectors *b* = *a*/2<011> (*a* is the lattice constant) which then progressively release the strain by gliding in {111} planes towards the interface. In the end, a straight 60° misfit dislocation (MD) terminated by two threading dislocations (TDs) is formed. The TDs, of screw orientation^6^, can make the MD further grow by gliding. At higher Ge contents, it has been shown that plastic relaxation is associated to the formation of edge MDs with Burgers vectors $b=\pm a/\mathrm{2[1}\bar{1}\mathrm{0]}$ or *b* = ±*a*/2[110] belonging to the interface plane. Edge MDs release twice as much misfit strain than 60° MDs. For a pure Ge film, a regular array of edge MDs can almost entirely release the film strain^7,8^. Typically this array is, however, not perfect and some TDs are also present which greatly deteriorate the film properties. In order to help develop methods for growing Ge films of high quality it is important to understand how the edge MDs form. This has been a topic of active research for decades and has been recently reviewed^9^.

The challenge is to find a mechanism accounting for the presence of edge MDs at the interface because they are not mobile and cannot nucleate at the surface and glide towards the interface. Furthermore, direct experimental observations of the nucleation and early stages of growth of edge MDs are not available due to the short time scales involved. Several mechanisms have been proposed^9^. The most credible ones rely on the nucleation of two complementary 60° MDs which are then combined to form an edge MD:

1 $$a/\mathrm{2[0}\bar{1}\bar{1}\mathrm{](1}\bar{1}\mathrm{1)}+a/\mathrm{2[101](1}\bar{1}\bar{1})\to a/\mathrm{2[1}\bar{1}\mathrm{0](001),}$$

In the so-called induced nucleation mechanism, a 60° MD first forms through the half-loop nucleation then favors the formation of a complementary 60° MD, both are combined at the interface^10–13^. In the cross-slip mechanism^9,14,15^, two orthogonal 60° MDs nucleate independently from each other. When the TD of one 60° MD reaches the other 60° MD, a cross-slip mechanism occurs and an edge MD is formed by further TD glide.

Since these mechanisms cannot be observed experimentally, atomic scale calculations can provide valuable insights. However such calculations are very challenging. Since the MDs are extended defects and the interaction between them is of long range, a very large number of atoms needs to be included in numerical simulations. The description of the interaction between the atoms must also be accurate, although unusual bonding configurations are present in the MDs core. Finally, the nucleation of MDs is an activated event involving thermal activation over a significant energy barrier and thus direct molecular dynamics simulations are not suited to reach the relevant time scales. A better strategy is to identify the minimum energy path for the rearrangements of the atoms which is optimal in the sense that it involves the lowest activation energy (the activation energy being defined as the difference in energy between the configuration of highest energy along a transition path and the initial configuration). This is also challenging because the number of atoms involved and the number of possible paths are very large.

Previous atomistic simulation studies have predicted activation energies larger than 10 eV for the nucleation of MDs in Ge/Si^16,17^. Such a high barrier cannot be overcome by thermal fluctuation on a short time scale even at a temperature close to the melting temperature. However these studies relied on empirical potentials whose accuracy for accounting correctly for dislocation core is highly questionable and they considered only a few possible mechanisms.

Here, we model and identify several mechanisms by using a state of the art global optimization approach based on the nudged elastic band (NEB) method for finding optimal minimum energy paths^18^. Furthermore, in order to have a reliable estimate of activation energy, we combine density functional theory (DFT) and Stillinger-Weber (SW) classical potential calculations. By comparison with DFT calculations on a dislocation dipole, we first quantify the error in dislocation core energy arising from using the classical SW potential. The energies calculated with SW during global optimization are then systematically corrected by this error after determination of the total length of the dislocations involved.

With our combined approach, we are able to identify new mechanisms for the nucleation of MDs which unlike those previously described have low enough activation energy to be activated under experimental conditions in the case of a pure Ge film. In particular, we find that for a highly strained film, a 60° MD most likely nucleates as a “split” half-loop with two branches of the loop gliding on separate planes. The threading dislocation of the 60° MD is oriented so as to lower the activation energy and enhance the probability of the nucleation of a complementary 60° MD. The two 60° MDs are then combined to form a 90° MD at the interface. This mechanism should dominate at an early stage of relaxation of the Ge film when the strain is high. The previously proposed cross-slip mechanism is more likely to be relevant at a later stage of film relaxation or in GeSi film with a low concentration of Ge.

## Results

### Dislocation core energy error from the SW potential

By comparison with DFT calculations, we quantify the error in dislocation core energy from the SW potential. These calculations are based on the assumption that both DFT and SW are able to properly estimate the elastic energy associated with a dislocation while only DFT can properly estimate the dislocation core energy. Details of the methods and systems used can be found in the Methods section.

The Table 1 shows the SW dislocation core energy error ${\rm{\Delta }}{E}_{{\rm{core}}}={E}_{{\rm{core}}}^{{\rm{SW}}}-{E}_{{\rm{core}}}^{{\rm{DFT}}}$ for different dislocation orientations and strains. We find that the SW potential significantly overestimates the dislocation core energy. While the error significantly depends on the orientation of the dislocation, the influence of the strain is small and unless stated otherwise will be neglected in further calculations. From this data we can correct the energies given by SW for large systems which cannot be modeled using DFT.

**Table 1:** Differences in dislocation core energy between SW and DFT calculation estimated for several orientations of the dislocation and for several uniform compressive strains.

strain (%)	Δ*E*_core_ (eV/Å)		
	screw	60	90
0	0.32	0.45	0.36
2	0.29	0.47	0.33
4	0.34	0.55	0.38

The SW potential is found to significantly overestimate the dislocation core energies.

### Nucleation of a 60° MD

We first discuss the nucleation of a 60° MD. We carried out global optimization of the transition path from a commensurate and defect-free pure 19 layers thick Ge film towards a configuration containing a straight 60° MD (with a $(a\mathrm{/2)[0}\bar{1}\bar{1}]$ Burgers vector) lying at the interface in the [110] direction. Note that the results presented for this specific dislocation can be extrapolated to other dislocations with Burgers vectors of the <101> family by symmetry operations so that equivalent results are expected for any 60° MD.

**Figure 1:**
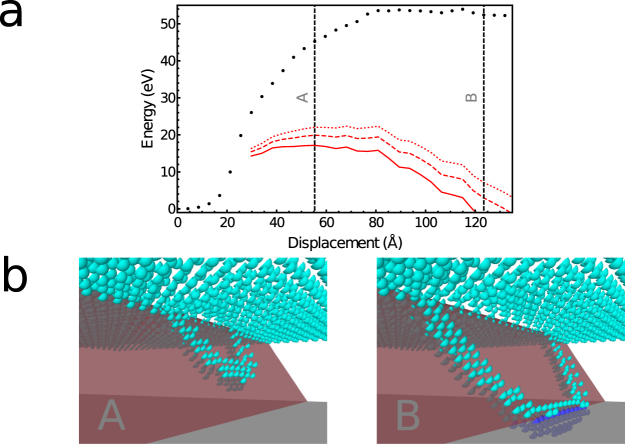
Illustration of the nucleation of a 60° MD through the half-loop mechanism. In (**a**), the corresponding energy profile is shown. The displacement corresponds to the length of the path in the 3 N dimensional space, where *N* is the total number of atoms. The energy is given with and without the dislocation core energy correction in red and black, respectively. The dotted, dashed and continuous lines are obtained by applying the correction with the smallest, average and largest values of ${\rm{\Delta }}{E}_{{\rm{core}}}^{{\rm{screw}}}$ and ${\rm{\Delta }}{E}_{{\rm{core}}}^{{\rm{60}}}$, respectively. The correction is applied only when the dislocation is clearly identifiable. The vertical dashed lines indicate the position along the path of the configurations A and B shown in (**b**). In (**b**), only the atoms in a local environment that does not correspond to that of a diamond lattice are shown. The light and dark blue atoms are Ge and Si atoms, respectively. The red plane is the $\mathrm{(1}\bar{1}\bar{1})$ plane.

In the standard nucleation mechanism, a half-loop dislocation forms from the surface in a {111} plane and expands by gliding as is illustrated in Fig. 1b. We were able to model this mechanism by using the NEB method starting from a linearly interpolated path with a relatively small number of intermediate images. However, it should be noted that the transition path obtained does not correspond to a minimum energy path since further relaxing the path with a larger number of intermediate images leads to a path where one TD has undergone a cross-slip. This cross-slip event is discussed in the next section.

Our calculations indicate that the half-loop dislocation is roughly composed of three straight segments: one screw TD, one 60° MD and one 60° TD. The corresponding energy profile is shown in Fig. 1a. Without any dislocation core correction the activation energy is larger than 50 eV.

Table 1 shows that the error in SW dislocation core energy depends on the strain. However, the strain is not homogeneous in the film and during the nucleation. It is therefore challenging to take into account the influence of the strain on the correction and we therefore neglect it by choosing fixed values for the corrections. The uncertainty arising from this choice is illustrated in Fig. 1a. The dotted, dashed and continuous red lines are obtained by applying the correction with the smallest, average and largest values of ${\rm{\Delta }}{E}_{{\rm{core}}}^{{\rm{screw}}}$ and ${\rm{\Delta }}{E}_{{\rm{core}}}^{{\rm{60}}}$, respectively. Figure 1a shows that the correction to the energy profile is much larger than the uncertainty. However, the latter remains quite large since the activation energy varies from 17 to 22 eV depending on the Δ*E* values. Furthermore, no correction is applied for dislocation kinks. Overall, we obtain a refined estimate of the activation energy which has to be considered as semi-quantitative. We find here that for a pure and perfect Ge film, the nucleation of a 60° MD is very unlikely to occur through the half loop dislocation mechanism since the activation energy is larger than 15 eV.

**Figure 2:**
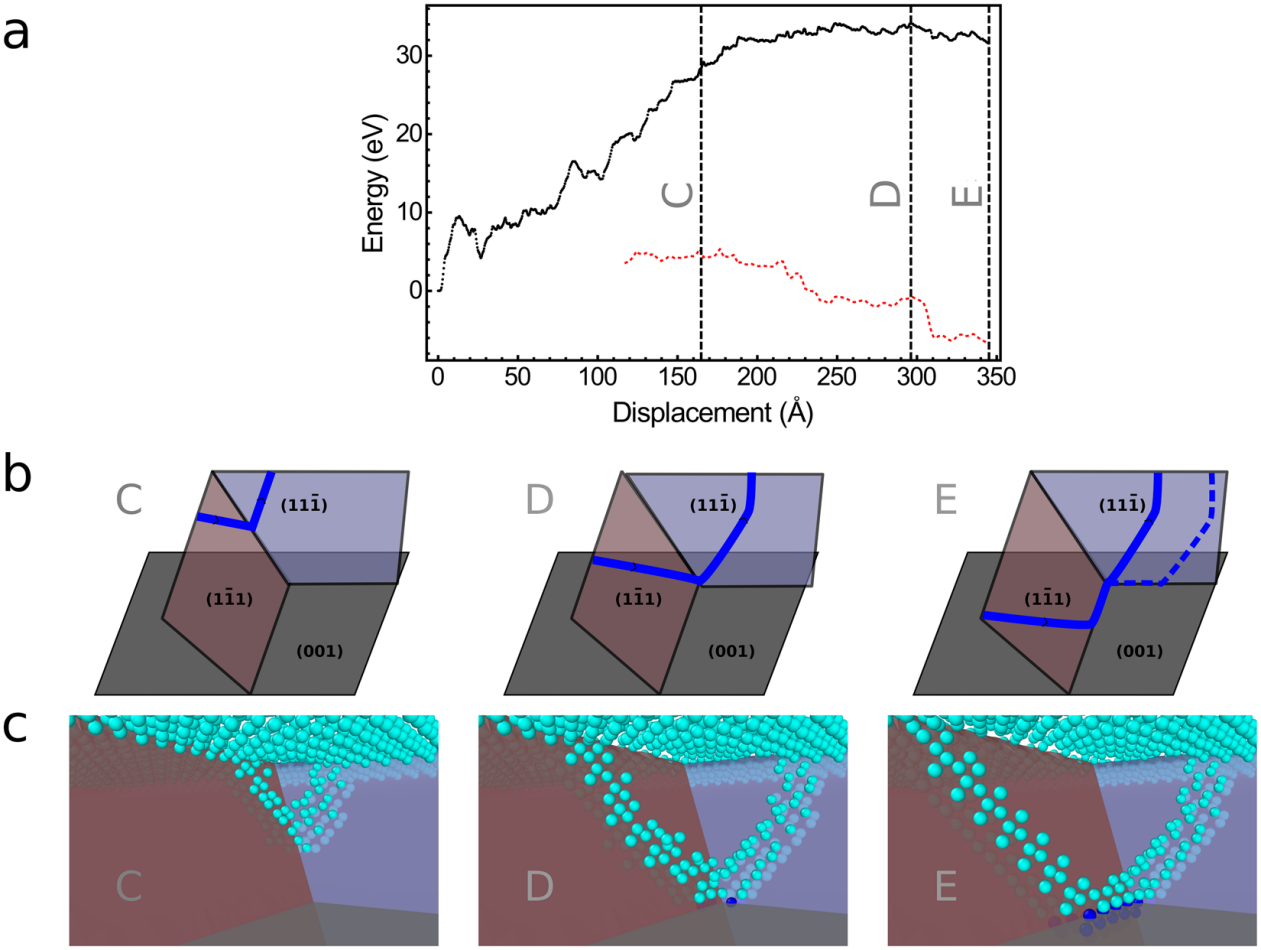
Illustration of the nucleation of a 60° MD from the “split” half-loop mechanism. In (**a**), the energy profile for the best transition path found for the nucleation of a 60° MD is shown. The energy is given with and without the dislocation core energy correction in red and black, respectively. The correction is applied only when the dislocation is clearly identifiable. The vertical dashed lines indicate the position along the path of the configurations shown in (**b**,**c**). In (**b**) the configurations are shown schematically. In **c**, only the atoms in a local environment that does not correspond to that of a diamond lattice are shown. The light and dark blue atoms are Ge and Si atoms, respectively.

However, the global optimization procedure allowed us to identify another mechanism having a much lower activation energy. Its energy profile is presented in Fig. 2a. This minimum energy path features a large number of intermediate minima. Most events connecting two subsequent intermediate minima are associated with either a double kink formation or annihilation, or kink diffusion. We will not study in detail these events here since we believe that the SW potential is not accurate enough at this scale. However, by correcting the dislocation core energy, we can get reliable information about the overall shape of the energy profile and estimate the activation energy of the process.

Three configurations (C to E) along the best identified path for the nucleation of a 60° MD are shown in Fig. 2b and c. Similarly to the standard half-loop mechanism, the dislocation nucleates from the surface as a half-loop. However, this one does not glide in a single plane. Rather, there are two branches gliding in separate planes. We call this mechanism the “split” half-loop dislocation nucleation. When the “split” half-loop grows, the leading front moves down along the line intersecting both glide planes. Once the half-loop reaches the interface, the MD can grow by glide of one TD (see configurations D to E in Fig. 2).

The corrected activation energy associated with this “split” half-loop nucleation is 6 eV. It is 14 eV lower than that of the standard half-loop dislocation nucleation. It is however too large to be overcome by thermal fluctuation which means that it must be activated by the presence of a defect. The presence of a step on the film can significantly lower the activation energy for the nucleation of a MD. The presence of a step indeed increases the volume of the region for which some strain is released by the nucleation of a dislocation, it can also act as a stress concentrator^19^ and finally, the step may be removed by the nucleation of dislocation^17^. Preliminary calculations indicate that the activation energy for the nucleation of a 60° MD can be lowered to 2 eV by the presence of a double D_B_ step.

### Orientation of threading arms of 60° MDs

The key difference between the standard and “split” half-loop mechanisms is the orientation of one threading arm. Indeed, the threading arm which is a screw dislocation in the standard half-loop mechanism becomes a 60° dislocation in the “split” half-loop mechanism. In previous studies, it was often assumed that threading dislocations of 60° MDs were screw dislocations. This assumption is based on experimental observation of screw threading dislocations for a Ge_0.32_ Si_0.68_/Si(001) low strain film^6^. However, for a pure Ge film with a larger strain our calculations clearly indicate that a 60° orientation is more favorable for the TDs.

**Figure 3:**
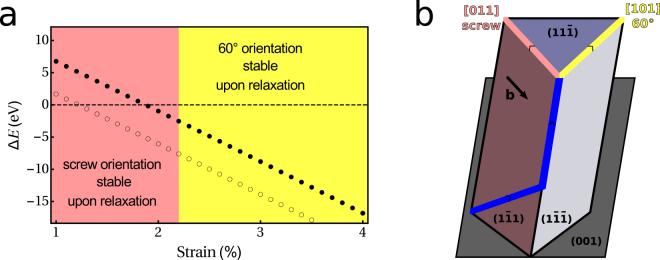
In (**a**), the difference in energy between a dislocation with a 60 (i.e. [101]) and screw (i.e. [011]) orientation for the TD is given as a function of the film strain. Filled and open circles are results without and with the dislocation core energy correction, respectively. The yellow and pink colors indicate region for which the 60 and screw are stable upon relaxation, respectively. In (**b**), the two orientations considered for the threading dislocations are illustrated.

In order to study the influence of the strain on the orientation of the TDs we consider the two configurations shown in Fig. 3b with a 60 Å long straight dislocation at the interface oriented in the [110] direction. For both configurations the TD going from the free surface to the interface is oriented in the $\mathrm{[10}\bar{1}]$ direction. The other TD is a 60° one (i.e. oriented in the [101] direction) in one configuration, and a screw one (i.e. oriented in [011] direction) in the other as shown in Fig. 3b. We apply a uniform biaxial strain to the system by varying the supercell size in directions parallel to the film. The difference in energy between both configurations is shown in Fig. 3a as a function of the film misfit strain (where the film misfit strain is defined as the strain in the film in the absence of dislocation). This energy difference is calculated without relaxing the system because upon relaxation, the [101] orientation is stable only for a strain larger than 2.2% whereas the [011] orientation is stable only for a lower strain (note that this threshold would most probably be shifted to a lower value if the dislocation core correction could be applied during the relaxation and not only as a post-treatment). The 60° orientation is more favorable at high and less favorable at low strains than the screw orientation.

We attribute this change of orientation to a competition between dislocation core energy and strain release. The TD changes its orientation from screw to 60° by glide in the $\mathrm{(11}\bar{1})$ plane thereby releasing extra strain. A 60° orientation of the TD is thus more efficient in releasing the film strain and is favoured for large film strain while at low film strain the screw orientation which must have a lower core energy is favoured.

### Nucleation of a 90° MD

#### Cross-slip mechanism

While expanding by gliding of its threading arm, a 60° MD may meet an orthogonal and complementary 60° MD. A screw threading arm can undergo cross-slip such that it thereafter glides in the glide plane of the complementary MD, which leads to the formation and growth of a 90° MD. This mechanism is known as the cross-slip mechanism. Although we did not model this mechanism, our study on the orientation of the TDs of 60° MDs provide valuable insights on the likelihood of this mechanism

At large strain, we found that the TDs of a 60° MD have a 60° orientation. Since a cross-slip can only occur for a screw dislocation, the TDs should first change their orientation to screw. According to Fig. 3 such a process has a high energy cost. Therefore, the cross-slip mechanism should be active only when the film strain is relatively low (i.e. for a Ge-poor GeSi film or at a later stage of relaxation of a Ge-rich GeSi film).

#### Induced nucleation of a complementary dislocation

**Figure 4:**
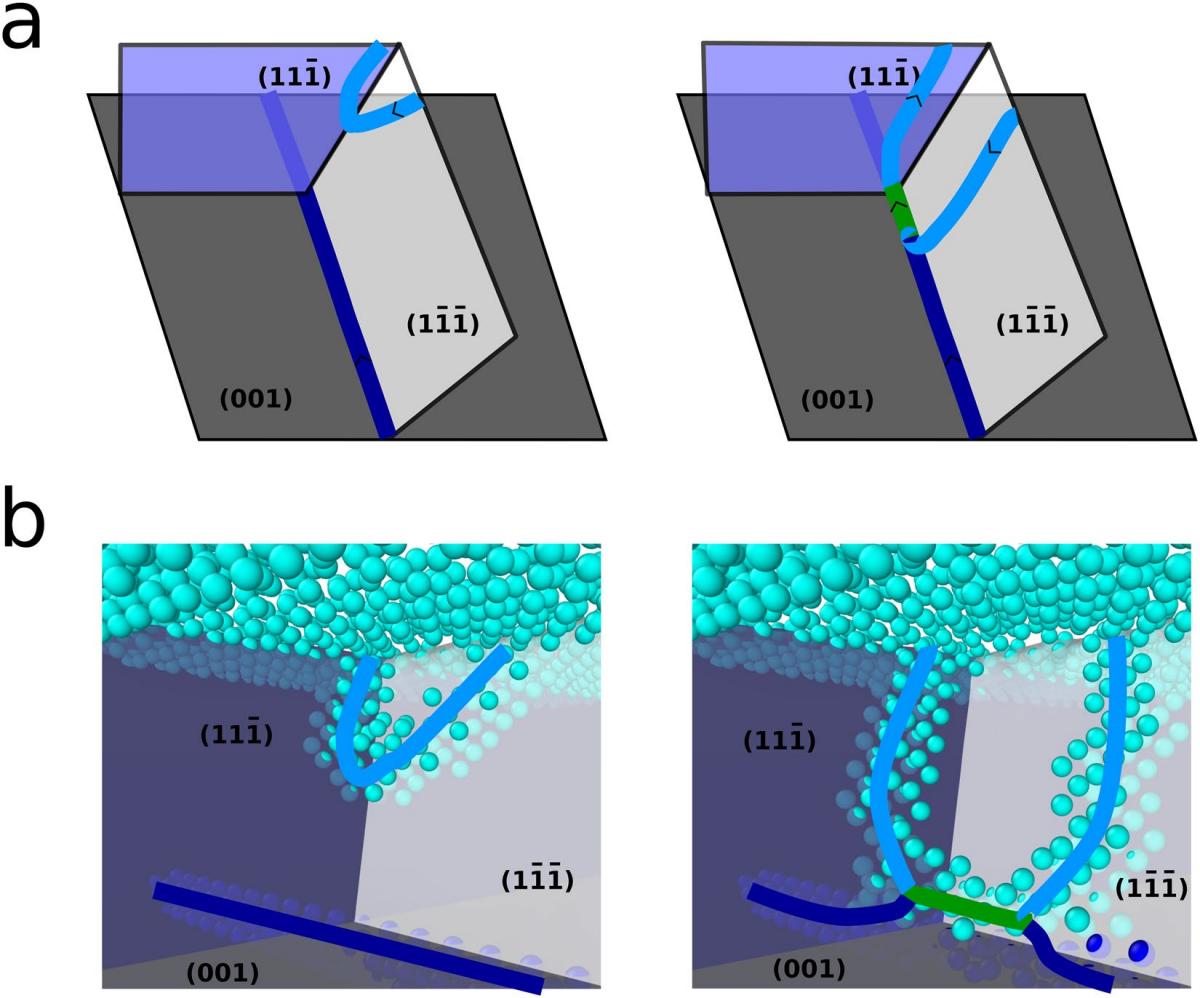
Illustration of the nucleation of a complementary dislocation in the presence of a straight 60° MD which ultimately leads to the formation of an edge dislocation. In (**a**), two successive configurations are shown schematically. In (**b**), the same configuration are shown by rendering only the atoms in a local environment that does not correspond to that of a diamond lattice. The light and dark blue atoms are Ge and Si atoms, respectively. The dark blue, light blue and green lines are the initial 60° MD, the complementary dislocation and the edge MD.

Previous studies have suggested that the presence of a straight 60° MD favors the nucleation of a complementary MD^9,11^. We carried out a global optimization of the transition path from a straight 60° MD with a $a\mathrm{/2[0}\bar{1}\bar{1}]$ Burgers vector to a straight 90° MD. As illustrated in Fig. 4, a complementary dislocation with Burgers vector *a*/2[101] can nucleate from the surface, glide in the $\mathrm{(1}\bar{1}\bar{1})$ plane and can be combined with the straight MD to form an edge MD according to Eq. (1). However, the corresponding activation energy is 7 eV which is similar to the activation energy for the nucleation of an isolated 60° MD. For a pure Ge film, a straight 60° MD at the interface does not favor the nucleation of a complementary MD.

**Figure 5:**
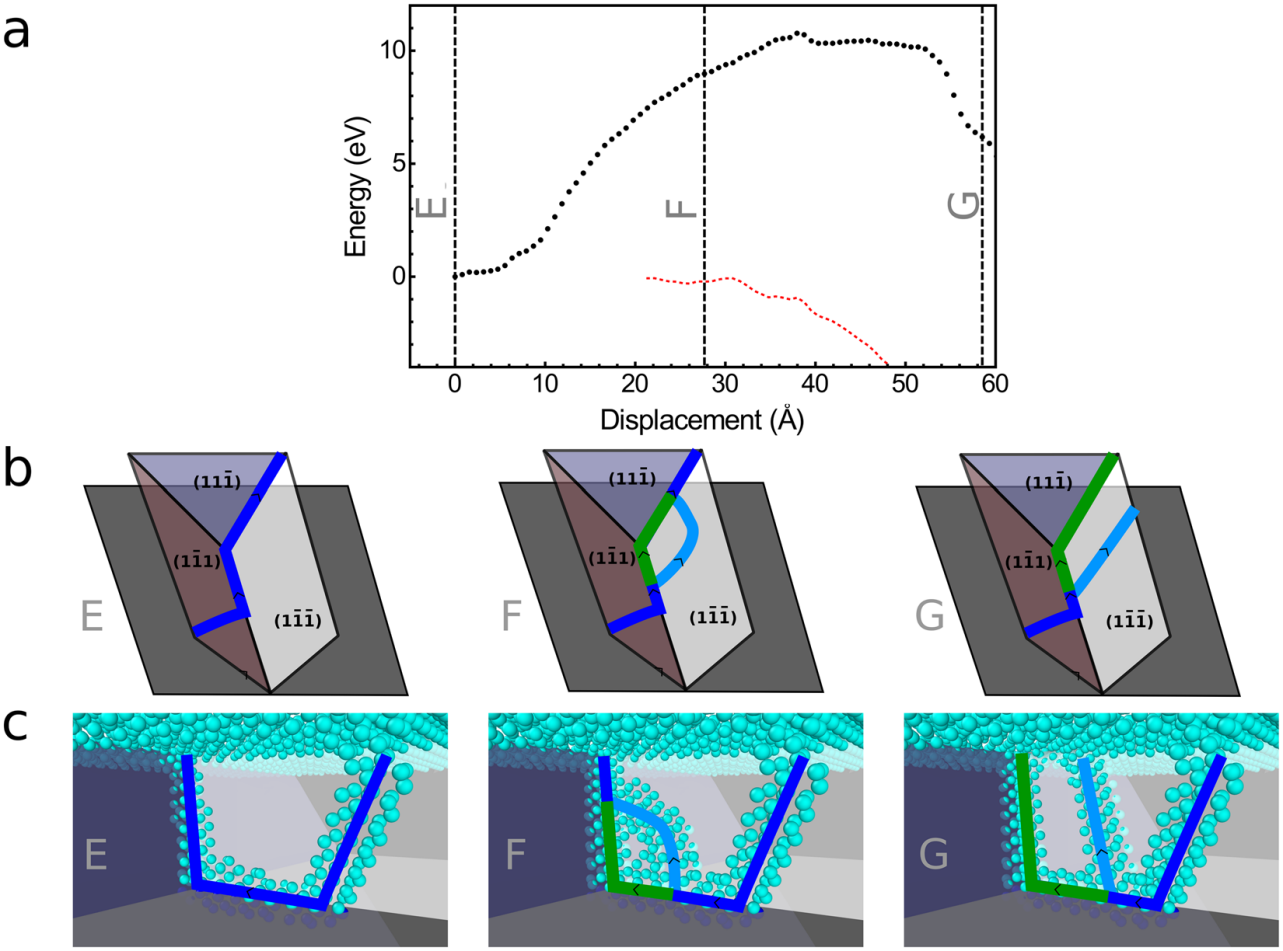
Illustration of the induced nucleation of an edge dislocation from a “split” half-loop dislocation. In (**a**), the energy profile for the best transition path found for the nucleation of a 90° MD starting from configuration E is shown. The energy relative to the energy of configuration E is given with and without the dislocation core energy correction in red and black, respectively. The correction is applied only when all dislocations are clearly identifiable. The vertical dashed lines indicate the position along the path of the configurations E, F and G shown in (**b**,**c**). In (**b**) the configurations are shown schematically. In (**c**), only the atoms in a local environment that does not correspond to that of a diamond lattice are shown. The light and dark blue atoms are Ge and Si atoms, respectively. The dark blue, light blue and green lines are the initial 60° MD, the complementary and the edge dislocations.

However, it turns out that a “split” half-loop dislocation can act as an efficient seed for nucleating a 90° MD. Such a dislocation, shown in configuration E in Fig. 2, has a $a\mathrm{/2[0}\bar{1}\bar{1}]$ Burgers vector. It can be combined with a complementary dislocation having a *a*/2[101] Burgers vector to form an edge MD following Eq. (1). This complementary dislocation glides in the $\mathrm{(1}\bar{1}\bar{1})$ plane. The “split” half-loop as shown in configuration E can strongly favor the nucleation of this complementary dislocation because one of its threading arms is oriented is the [101] direction and belongs to the glide plane of the complementary dislocation. Therefore, as illustrated in Fig. 5b and c, the complementary dislocation can nucleate close to the junction between the TD and the MD and is directly combined with the first MD to form an edge dislocation. The corresponding energy profile is shown in Fig. 5a. We cannot provide a quantitative estimate of the corresponding activation energy since the correction to dislocation core energy can be applied only when the dislocations are clearly identifiable and since the corrected energy is already lower than the initial energy once the dislocation is clearly identifiable. However, it is clear that the activation energy should be either very small or that this transition might even be spontaneous. It is then very likely that a 90° MD may be created spontaneously when a 60° MD forms by the “split” half-loop mechanism.

## Summary and Discussion

We have studied several mechanisms for the formation of 60° and 90° MDs. We find that depending on the orientation of TDs of 60° MDs different mechanisms should govern the plastic relaxation of a GeSi/Si film. Since the orientation of TDs depends on the strain, we can conclude that the mechanisms governing plastic relaxation will also depend on the film strain. Overall, our calculations indicate that plastic relaxation in GeSi/Si(001) should follow the following scenario:

At high film strain (i.e. at an early stage of the plastic relaxation of a Ge rich GeSi/Si(001) film), plastic relaxation starts with the nucleation of a 60° MD by the “split” half-loop mechanism with both branches of the half loop gliding in different planes. This nucleation is activated by the presence of steps. Since during the growth of the film, steps should be moving on the surface these “split” half-loops can nucleate all over the film. At an early stage of the expansion of the “split” half-loop, they induce the nucleation of complementary dislocations which immediately lead to the formation of an edge MD as illustrated in Fig. 5.

At lower film strain (i.e. at later stages of the plastic relaxation of a Ge rich GeSi/Si(001) film or during the relaxation of a Ge poor GeSi/Si(001) film), TDs of 60° MD are screw dislocations and 60° MDs nucleate through the half-loop mechanism. This nucleation is also activated by the presence of defects. At low strains, steps are apparently not sufficient to initiate the formation of 60° MDs since TEM experiments clearly show that 60° MDs nucleate relatively sparsely^6^. The presence of a 60° MD may help but is not sufficient to induce the nucleation of a complementary dislocation. The main mechanism for the formation of edge MDs is then probably the cross-slip mechanism, possible in that case since TDs are screw dislocations.

Overall, this study provides the first explanation of why the proportion of 90° MDs increases with Ge content^20–22^. Hopefully, this theoretical study will stimulate additional experimental works. Our prediction could be experimentally confirmed by observing the change in orientation of TDs of 60° MDs. This is however a challenging task since this change of orientation at high film strain strongly favours the nucleation of a 90° MD so it would only be transitional and hard to observe. The key might be to work at intermediate strain (large enough to activate the cross-slip but small enough so that the induced nucleation of 90° MD would not be spontaneous).

## Method

### System

A strained layer of Ge on a Si(001) substrate is modeled atomistically. In order to take into account the long range strain field of dislocations we consider a system containing 80 000 atoms. The dimensions of the supercell along the *x* ($\mathrm{[1}\bar{1}\mathrm{0]}$) and *y* ([110]) directions are 153.6 × 153.6 Å^2^ and are set according to the Si lattice constant. Periodic boundary conditions are applied along both the *x* and *y* directions. The influence of the supercell size is discussed in detail in the supplementary material. We find that further increase of the system size would not change significantly the results shown. For instance, the difference in activation energy for the “split” half loop mechanism is less than 0.3 eV when doubling the system size along both the *x* and the *y* directions. The substrate contains 31 layers of Si in the *z* ([001]) direction. The two bottom layers are kept fixed to represent constraints due to the bulk which is not explicitly included in the simulations. The coherent Ge film is 19 layers thick with a *p*(2 × 1) dimer reconstruction of the surface.

### Calculating the SW dislocation core energy error

The first challenge for carrying out atomistic calculations is to correctly describe the atomic interactions within a large system containing a very large number of atoms. Our calculations rely on the Stillinger-Weber (SW) potential^16,23,24^, which efficiently reproduces the elastic properties of Ge and Si. However, this potential was not parameterized using dislocations in the training set and although it was found to reproduce most of the dislocation core configurations predicted by DFT^25,26^, for Si, it was shown to be qualitatively wrong when predicting the relative energies of known core configurations^26,27^.

We consider model systems containing a dislocation dipole (i.e. a pair of straight dislocations with opposite Burgers vector) with full periodic boundary conditions. The energy of the dipole can be written as:

2 $${E}_{{\rm{dipole}}}={n}_{{\rm{at}}}{E}_{{\rm{coh}}}+2l{E}_{{\rm{core}}}+{E}_{{\rm{strain}}},$$

where *n*_at_ is the number of atoms in the system, *E*_coh_ is the cohesive energy, *l* is the length of one dislocation, *E*_core_ is the dislocation core energy, and *E*_strain_ is the energy due to the strain deformation induced by the dislocation dipoles and its elastic interaction with periodic images. We assume that *E*_strain_ is estimated accurately by both the SW potential and by DFT such that ${E}_{{\rm{strain}}}^{{\rm{SW}}}={E}_{{\rm{strain}}}^{{\rm{DFT}}}$, where the superscript indicates the method used for calculating the energy. By calculating the dipole energy and the cohesive energy with both SW and DFT and using Eq. (2), we can estimate the error in the dislocation core energy given by SW as:

3 $${\rm{\Delta }}{E}_{{\rm{core}}}={E}_{{\rm{core}}}^{{\rm{SW}}}-{E}_{{\rm{core}}}^{{\rm{DFT}}}=\frac{1}{2l}({E}_{{\rm{dipole}}}^{{\rm{SW}}}-{E}_{{\rm{dipole}}}^{{\rm{DFT}}}-{n}_{{\rm{at}}}({E}_{{\rm{coh}}}^{{\rm{SW}}}-{E}_{{\rm{coh}}}^{{\rm{DFT}}}))\mathrm{.}$$

More details about the calculations are given in the supplementary material. We also check the effect of imposing a biaxial strain. The error in the dislocation core energy is found to be only slightly dependent on the strain. The estimated error is shown in Table 1.

### DFT correction to SW energy

The SW energy of a configuration containing dislocations of length ${l}_{{x}^{\circ }}$ with a *x* ^°^ orientation is corrected using:

4 $${E}_{{\rm{corrected}}}={E}^{{\rm{SW}}}+\sum _{x}{l}_{{x}^{\circ }}{\rm{\Delta }}{E}_{{\rm{core}}}^{{x}^{\circ }}$$

In our calculations, most dislocations either have a screw, 60° or edge orientation such that we can directly use values given in Table 1. For other orientations, we use a linear interpolation as follows:

5 $${\rm{\Delta }}{E}_{{\rm{core}}}^{{x}^{\circ }}=(\begin{array}{cc}\frac{x}{60}{\rm{\Delta }}{E}_{{\rm{core}}}^{60}+\frac{60-x}{60}{\rm{\Delta }}{E}_{{\rm{core}}}^{{\rm{screw}}} & ,\,x\, < \,\mathrm{60;}\\ \frac{x-60}{30}{\rm{\Delta }}{E}_{{\rm{core}}}^{{90}^{\circ }}+\frac{90-x}{30}{\rm{\Delta }}{E}_{{\rm{core}}}^{{60}^{\circ }} & ,\,x\, > \,60.\end{array}$$

We neglect the influence of the strain for Δ*E*_core_ and unless stated otherwise use the average value.

Note that the DFT correction cannot be made in the very early stage of the dislocation nucleation but only once the dislocation is clearly identifiable.

### DFT calculations

DFT calculations are carried out using GPAW^28,29^, with the PBE exchange-correlation functional^30^ and a real-space grid with a 0.2 Å spacing. Six *k*-points are used in the direction parallel to the dislocation line. The supercell length in that direction is equal to the Burgers vector length. The configurations are relaxed until the maximum force on an atom is smaller than 0.02 eV/Å.

### Global optimization of transition path

We carried out a global optimization of the transition path for the formation of 60° and 90° MDs. In semi-conductor materials, the transition path corresponding to the nucleation of a dislocation contains several local energy minima separated by energy barriers^18^. The key is then to find the good intermediate minima.

Our strategy which was described in^18^ aims at finding the best transition path by finding the best intermediate states for the path. New intermediate configurations are generated using heredity transformations from known configurations. Such a transformation consists in selecting coordinates of some of the atoms from one of the parent configurations (for instance by taking all the atoms which are inside a region defined by specifying two planes) while the coordinates of the remaining atoms are selected from the other parent configuration. Paths between newly generated configurations are relaxed with the help of a stabilized nudged elastic band (SNEB) method until the norm of the 3*N*-dimensional force vector for each image has dropped below 0.02 eV/Å. The SNEB method is a variant of the nudged elastic band method^31–33^ with improved efficiency when working with many replicas and/or a poor resolution. It is introduced in detail in the supplementary material document.

A large number of continuous transition paths between an initial and a final configurations are obtained by connecting the intermediate configurations found. We then select the intermediate configurations which give the lowest activation energy for the overall transition.

The energy profiles of the transition paths obtained by global optimization on the SW potential energy profile are then corrected by determining the dislocation length and rectifying the dislocation core energy as described in the core of this study.

### Data availability

The datasets generated during and/or analysed during the current study are available from the corresponding author on reasonable request.

## Electronic supplementary material

Supplementary Information
