# Supplementary material for: Atomic Scale Formation Mechanism of Edge Dislocation Relieving Lattice Strain in a GeSi overlayer on Si(001)
Source: Sci Rep. 2017 Sep 20;7:11966. doi: 10.1038/s41598-017-12009-y (PMC5607354; doi:10.1038/s41598-017-12009-y)
Supplement: Supplementary file 1 — Supplementary Information [file 41598_2017_12009_MOESM1_ESM.pdf]

Atomic Scale Formation Mechanism of Edge Dislocation Relieving Lattice Strain in a GeSi overlayer on Si(001)

E. Maras[1,2,*], L. Pizzagalli[3], T. Ala-Nissila[1,2,4,5], and H. Jónsson[2,6]

[1]COMP Center of Excellence Aalto University School of Science, FI-00076 Aalto, Espoo, Finland
[2]Department of Applied Physics, Aalto University School of Science, FI-00076 Aalto, Espoo, Finland
[3]Department of Physics and Mechanics of Materials, Institut Pprime, CNRS UPR 3346, Université de Poitiers, SP2MI, BP30179, 86962 Futuroscope Chasseneuil, France
[4]Department of Physics, Box 1843, Brown University, Providence, RI 02912-1843, U.S.A.
[5]Departments of Mathematical Sciences and Physics, Loughborough University, Loughborough, Leicestershire LE11 3TU, United Kingdom
[6]Faculty of Physical Sciences, University of Iceland, 107 Reykjavík, Iceland
[*]e1000.3000@gmail.com

ABSTRACT

In this supplementary information we study the influence of the supercell size on our results. We also provide more information on the DFT correction to the Stillinger Weber estimate of the energy. Finally, we detail the stabilized nudged elastic band method.

1 Supplementary material

S1.1 Influence of supercell size

It is well known that dislocations have a long range strain field such that in atomistic calculations with periodic boundary conditions one dislocation can interact with its periodic images and the energetic may significantly depends on the system size considered. In this section, we discuss the influence of the system size on the results presented in the main manuscript.

S1.1.1 Nucleation of 60° MD

Our calculations indicate that a 60°MD nucleates most efficiently through a broken half-loop mechanism. Fig. S1 shows the corresponding energy profile obtained with a 154Å × 154Å and a 308Å × 308Å simulation cell. We find that the difference in energy is very small. The activation energy calculated for both system size (after correction of the dislocation core energy) differ by less than 0.1 eV. In the early stage of the nucleation shown in Fig. 2 (of the main paper) the broken half-loop is small compared with the system size. It can then be considered as a point defect and its strain field decreases as $1/r^3$ where r is the distance from the defect[2]. The maximum value of the strain field ε_{xx} at the boundary of the system is less than 0.003 for the 154Å × 154Å system and 0.0004 for the 308Å × 308Å system.

S1.1.2 The case of an infinite straight 60° MD

We now consider an infinite 60°MD at the interface between the film and the substrate. The dislocation is oriented in the y direction and the free surface is normal to the z direction. The dislocation is at position $x = 0$. We consider periodic boundary conditions in the x and y direction. The cell size in the x direction is given by L_x. The film thickness is noted L_z. Let $\varepsilon_{xx}^{L_x}(x,z)$ be the strain field due to one such dislocation and $\varepsilon_{xx}^{\infty}(x,z)$ being the limit of $\varepsilon_{xx}^{L_x}(x,z)$ when $L_x \to \infty$. The average strain in the film due to the dislocation in the finite system is

$$\overline{\varepsilon_{xx}^{L_x}} = \frac{1}{L_x L_z} \int_0^{L_z} \int_{-L_x/2}^{-L_x/2} \varepsilon_{xx}^{L_x}(x,z)dxdz \tag{S1}$$

Due to the periodic boundary condition, $\int_x \varepsilon_{xx}^{L_x}(x,z)dx = -\mathbf{b}.\mathbf{u_x} = \mathbf{b/2}$ where $\mathbf{u_x}$ is a unit vector in the x direction. Equation S1 is then simplified to :

$$\overline{\varepsilon_{xx}^{L_x}} = -\frac{b}{2L_x} \tag{S2}$$

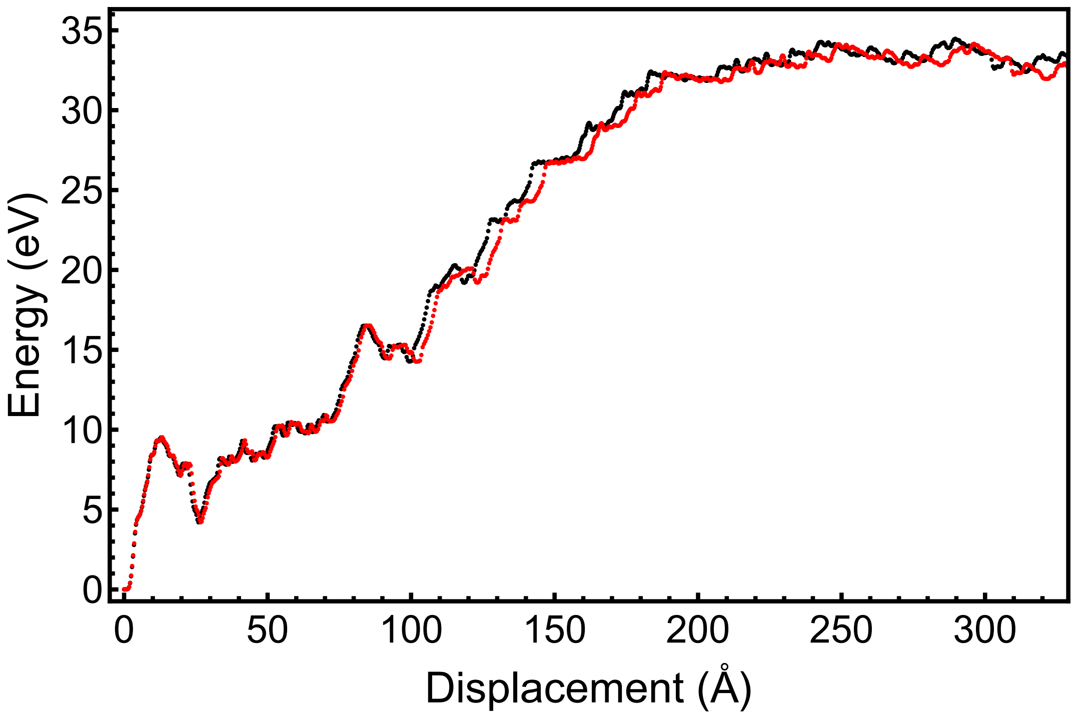

Figure S1. Energy profile for the nucleation of a 60°MD through the split-half loop mechanism calculated with a supercell size of 154Å × 154Å (black) and 308Å × 308Å (red).

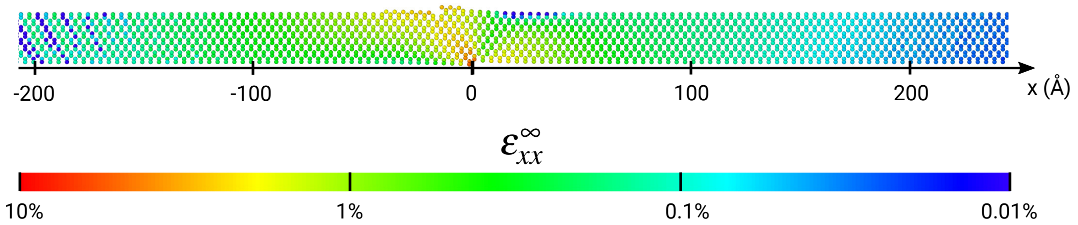

Figure S2. Strain field ε_{xx} in the film due to a 60°MD.

Let $\overline{\varepsilon_{xx}^{\infty}}^{L_x}$ be the strain in the infinite system averaged over the region $x \in [-L_x/2, +L_x/2]$. We have

$$\overline{\varepsilon_{xx}^{\infty}}^{L_x} = \int_0^{L_z} \int_{-L_x/2}^{-L_x/2} \varepsilon_{xx}^{L_x}(x,z)dxdz \tag{S3}$$

$\Delta\varepsilon_{xx}(L_x) = \overline{\varepsilon_{xx}^{L_x}} - \overline{\varepsilon_{xx}^{\infty}}^{L_x}$ gives an estimate of the average strain error due to the use of a finite size cell. We numerically compute the strain field of a 60°MD in a cell with $L_x = 1700$Å and associated it with the strain field of an infinite system. The strain field obtained around the MD is shown in Fig. S2. From this, we compute $\Delta\varepsilon_{xx}(L_x = 154\text{Å}) \simeq 0.3\%$. To the first order, the effect of periodic boundary condition on the strain field in the film was to increase the average film strain by 0.3% as compared with an infinite system.

S1.1.3 Influence on the change of TD orientation

In the main manuscript we modeled a half-loop with 2 different orientations for one TD. The system size used in our calculation was 154Å × 308Å. The change of orientation of the TD can be described by adding a dislocation half loop as illustrated in Fig. S3. This added half-loop is small compared with our simulation cell and it can be associated with a point defect (as far as we are concerned with the interaction with periodic images) and the influence of periodic images can be neglected.

However, the dependence on system size of the strain around the dislocation half-loop should not be neglected since the length of the MD is not negligible with respect to the supercell size. The contribution of periodic images should be similar but smaller than that of an infinite 60°MD. Fig. S4 shows the variation of the difference in energy between both orientations of the TD as a function of film strain calculated for both a 154Å × 308Å and a 308Å × 308Å supercells. We find that the curve is

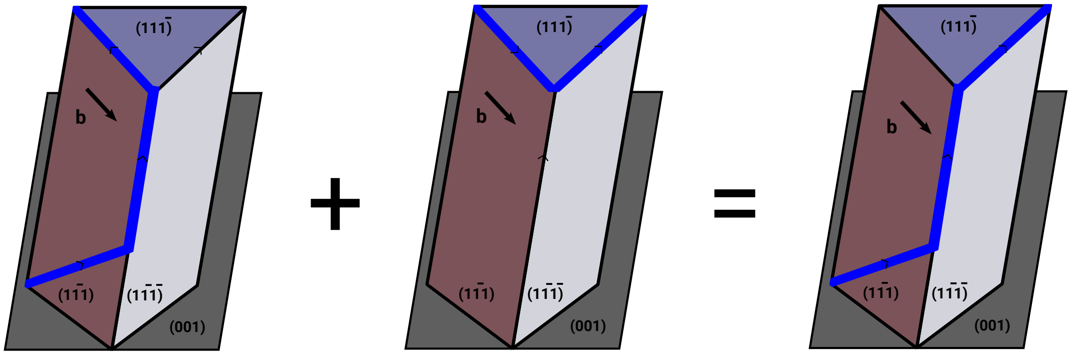

Figure S3. The change of orientation of a TD can be seen as the addition of a dislocation half-loop.

shifted to the left by around 0.2 %. We can infer from the previous section that this shift cannot be larger than 0.3% when increasing the system size L_x toward infinity.

S1.1.4 Induced nucleation of an edge MD

The nucleation of a 90°MD is illustrated in Fig. 5. This process can be divided in two parts. First a loop of complementary 60°MD nucleates and a part of this loop is combined with the initial dislocation to form a 90°MD. During this stage, the nucleating loop is small compared with our supercell size. It can therefore be considered as a point defect and the dependence of the energetics on the system size should be negligible. In the second stage, the 90°MD grows by glide of the complementary 60°MD. The extended defect can no longer be considered as a point defect and the system size can then influence the energetics. However, the growth of the 90°MD is so favorable that this effect should be negligible.

S1.2 DFT correction to the SW estimate of the energy of dislocations in Ge

The SW potential function overestimates the energy of dislocations in Ge and Si because it is designed to describe primarily fourfold coordination of atoms and tetrahedral arrangements of the bonds to the neighboring atoms. Si and Ge atoms can, however, have other coordinations without as high penalty in energy as the SW potential function predicts. The error in the SW dislocation energy is mainly due to incorrect description of the dislocation core where bond angles and even coordination deviate from the ideal values. DFT can give more accurate estimates of the dislocation energy, but the size of the simulation systems needed to represent dislocations are too large for DFT calculations. However, since the main error in the SW estimate comes from dislocation cores, a DFT estimated correction to the SW dislocation energy can be obtained by comparing SW and DFT results on smaller systems.

The error in the SW estimate of the dislocation core energy was calculated for three dislocation orientations: Screw, 60° and 90°. Systems containing two complementary dislocations were constructed including 276 or 288 atoms depending on the orientation of the dislocations. In such small systems, the two complementary dislocations may glide towards each other and annihilate so special care needs to be taken in some of the calculations, as described below.

A 60°dislocation has only one easy glide plane. Then, a supercell can be chosen as shown in Fig. S5a to prevent the dislocations from gliding towards each other when a local minimization of the the energy is carried out. The calculation of the energy of the dislocation was carried out using both SW and DFT and a difference of $\Delta E^{60^\circ}_{\text{core}} = 0.45$ eV/Å was obtained. This then provides a correction to the SW calculated dislocation energy in the large system.

For other dislocations, such as the screw dislocation, there are additional easy glide planes and a system cannot be chosen in such a way as to prevent the complementary dislocation pair from annihilating during energy minimization. Only a more restrictive minimization of the energy can then be made. It is carried out in two steps. First, the energy is minimized only with respect to coordinates of atoms outside the dislocation cores while keeping the core atoms fixed. Then, atoms outside the core are kept fixed and the energy minimized with respect to the core atoms. Core atoms were defined as atoms located within 8 Å of the center of the dislocation. In order to check the accuracy of this latter method the correction to the energy of the 60°dislocation was calculated again using the system shown in Fig. S5b. The results were similar to those obtained with the first method, 0.41 eV/Å, only a 10% difference.

The screw dislocations have two easy glide planes in the cubic diamond structure. The two step restrictive minimization of the energy was therefore used to estimate the DFT correction to the SW estimate $\Delta E^{screw}_{\text{core}}$.

The 90°dislocations are sessile so the calculation of $\Delta E^{90^\circ}_{\text{core}}$ can be obtained using local minimization of the energy with respect to all atom coordinates.

The calculated DFT correction to the SW dislocation core energy for screw, 60° and 90° dislocations for several values of uniform compressive strain are given in Table 1 in the main text.

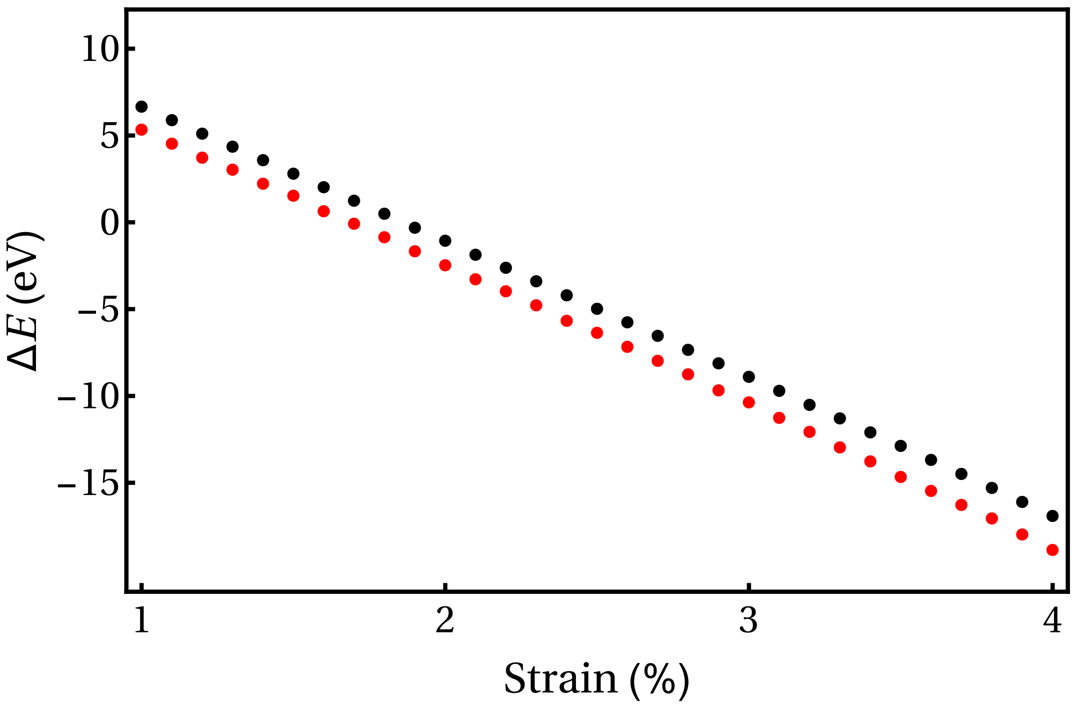

Figure S4. Difference in energy between a dislocation with a 60°(i.e. [101]) and screw (i.e. [011]) orientation for the TD is given as a function of the film strain. Calculation were carried out with a supercell size of 154Å × 154Å (black) and 308Å × 308Å (red)

Below is a brief description of the atomic configurations of the dislocation cores of the various dislocations.

S1.2.1 Screw dislocation

In both the DFT and SW calculations presented here, the screw dislocation cores adopt the configuration predicted by Hornstra[1]. This has been designated as configuration A[3–5]. Another configuration involving a double period reconstruction along the dislocation line, turns out to be more stable[3]. It has been labeled as C_2[4,5]. DFT calculations for Si give 0.15 eV/Å lower energy for C_2 than A cores[3,4]. But, the C_2 configuration is less mobile than the A configuration[5] so the A configuration is more relevant for the nucleation process.

S1.2.2 60° dislocation

The dislocation core obtained for the 60° dislocation in Ge is similar to the S2 configuration[4,5] obtained for Si except that weak bonds are not present in the core. For Si, it has been estimated that the S3 and G configurations have 0.21 eV/Å and 0.36 eV/Å

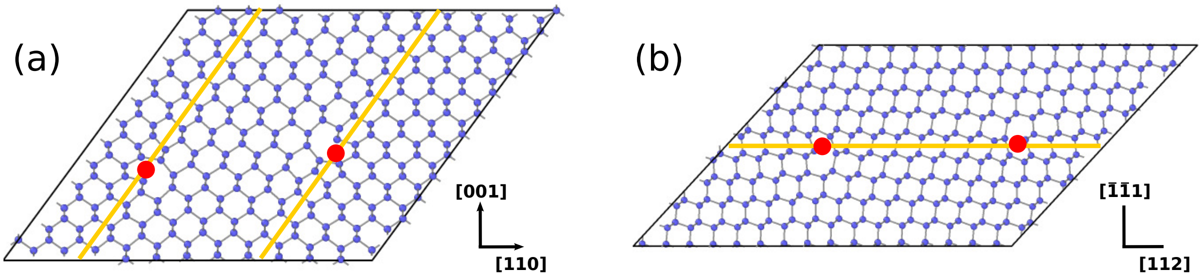

Figure S5. A pair of complementary 60° dislocations. The coordinates of the atoms, shown in blue, were obtained by locally minimizing the energy in DFT calculations. Red circles indicate dislocation cores. Orange lines indicate a gliding plane for the dislocations. In the simulation cell shown in (a) the dislocations cannot glide towards each other and annihilate, but in (b) they can.

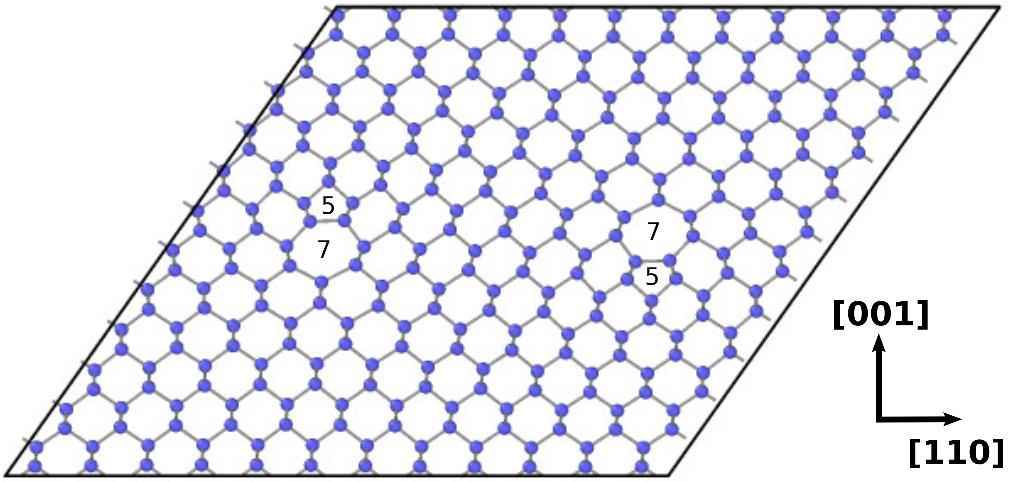

Figure S6. A pair of complementary edge dislocations. The coordinates of the atoms, shown in blue, were obtained by locally minimizing the energy in DFT calculations. The dislocation core consists of five and seven atom rings.

lower energy than the S2 configuration[5]. However, the S3 and G configurations are sessile and are not expected to be present during the nucleation of dislocations[4].

S1.2.3 90° dislocation
Figure S6 shows the configuration of two complementary 90°edge dislocation after minimization of the energy obtained from DFT calculations. The core consists of five and seven atom rings[1,6,7].

S1.3 Stabilized nudged elastic band method
The NEB method[8,9] is widely used to find minimum energy paths (MEPs) for transitions such as chemical reactions and diffusion events[10]. Typically, such calculations involve on the order of 100 to 1000 atoms with only a few of the atoms moving significantly during the transition and the paths have at most a few maxima in the energy along the path. The application here to nucleation and displacements of dislocations in a covalently bonded system where a large number of atoms move during the transition and the path involves a large number of maxima brings the NEB method into a new and more challenging application area. During our studies of the Ge/Si system presented here, we have found it necessary to modify the method to improve its stability and efficiency.

An NEB calculation is initiated by specifying some path between given endpoints which are usually taken to be local minima on the energy surface corresponding to initial and final states of the transition. The path is discretized by creating a set of N configurations, i.e. 'replicas', of the system and the task of the NEB calculation is to bring the intermediate replicas to the nearest MEP. The location of replica i is specified by a vector $\boldsymbol{R}_i$ consisting of the coordinates of all the atoms in the system. By choosing N to be large enough, the resolution of the path, characterized by the average distance $\bar{d}$ between adjacent replicas, can be made fine enough. Typically, the required resolution corresponds to ca. 5 replicas per maximum along the path but it is also affected by the curvature of the MEP. The paths studied here in the Ge/Si system are much longer, on the order of hundreds of Å, and involve many more maxima in the energy than typical applications of the NEB method. The energy along the paths often has hundreds of maxima because the transition mechanism involves sequential displacements of different groups of atoms. However, in order to keep the computational effort manageable, the number of replicas is chosen here to be as small as possible, so the resolution is smaller than in a typical NEB calculation. Two modifications of the NEB method were implemented to ensure stability and proper convergence of the calculations. We will refer to this modified version as stabilized nudged elastic band (SNEB) method.

In the commonly used NEB method, the force acting on a replica is given by

$$\boldsymbol{F}_i = -\nabla V(\boldsymbol{R}_i) + (\nabla V(\boldsymbol{R}_i)\cdot\hat{\boldsymbol{\tau}}_i)\hat{\boldsymbol{\tau}}_i + \boldsymbol{F}^s_{i\|}, \tag{S4}$$

where $\hat{\boldsymbol{\tau}}_i$ is the tangent to the path and the first two terms on the right hand side represent the perpendicular component of the true force - the force arising from the atomic interactions - while the last term represents an additional spring force introduced

to maintain equal spacing between the replicas along the path. The spring force only acts in the direction of the path and is given by

$$\boldsymbol{F}^s_{i\|} = k\,(d_i - d_{i-1})\,\hat{\boldsymbol{\tau}}_i, \tag{S5}$$

where k is the spring constant and $d_i = |\boldsymbol{R}_{i+1} - \boldsymbol{R}_i|$ is the distance between replicas i and $i+1$.

An important issue is the estimate of the local tangent to the path, $\hat{\boldsymbol{\tau}}_i$. In early implementations of the NEB method[8,9] the tangent at replica i was estimated from the line segment between replicas $i+1$ and $i-1$. In some applications instabilities in the path were found to occur, in particular for transitions in covalent systems such as Si. Kinks where the tangent changed abruptly from one replica to another could develop on the path and keep oscillating as optimization iterations were carried out, preventing convergence of the calculations. One way to remedy this was to include some part of the perpendicular component of the spring force so as to straighten out the path and eliminate the kinks[9]. This component of the spring force was turned on when the angle between the line segments $\boldsymbol{R}_i - \boldsymbol{R}_{i-1}$ and $\boldsymbol{R}_{i+1} - \boldsymbol{R}_i$ became large. However, by using a more stable estimate of the tangent, obtained from the line segment from replica i to the adjacent replica of higher energy[11], kinks were largely avoided in most applications without including the perpendicular spring component.

In the present application, were the number of replicas is rather small relative to the number of energy maxima along the path, and where the path can have large curvature, we find that it is again useful to include a scaled perpendicular component of the spring force in order to ensure convergence, even though the more stable tangent estimate[11] is also used. The force on an image then has an additional contribution, $\boldsymbol{F}^s_{i\perp}$, to the force given in eqn. (S4) where

$$\boldsymbol{F}^s_{i\perp} = kf(\phi_i)\,[\Delta\boldsymbol{R} - (\Delta\boldsymbol{R}\cdot\hat{\boldsymbol{\tau}}_i)\hat{\boldsymbol{\tau}}_i]\,. \tag{S6}$$

Here, $f(\phi_i)$ is a switching function that goes smoothly from zero if the path is straight to unity if adjacent segments of the path form a right angle and $\Delta\boldsymbol{R} = (\boldsymbol{R}_{i+1} - \boldsymbol{R}_i) - (\boldsymbol{R}_i - \boldsymbol{R}_{i-1})$. The switching function is chosen to be

$$f(\phi_i) = \frac{1}{2}\,(1 + \cos(\pi\cos(\phi_i)))\,. \tag{S7}$$

The perpendicular spring force was also included in preliminary calculations of this system and was found to significantly improve the convergence of the NEB calculations[12].

The second modification of the NEB method used here relates to the way in which an equal spacing is maintained between the replicas during the optimization of the path. While the spring force, $\boldsymbol{F}^s_{i\|}$, suffices in typical NEB calculations involving 5 to 10 replicas, it is not efficient enough for paths with hundreds of replicas. The reason is that the spring force acting on each replica only depends on the difference in distance to the two adjacent replicas. As a result, a small difference in spacing between replicas can build up along the path and result in significant variation in the spacing from one region of a long path to another. An NEB calculation is generally considered converged when the norm of the force on a replica is smaller than a given value, F_{conv}. This convergence criterion ensures that $|\boldsymbol{F}_i| \leq F_{\mathrm{conv}}$ which means that $d_i - d_{i-1} \leq F_{\mathrm{conv}}/k$. If there is a systematic variation in the distances between replicas, then this only guarantees $d_{N-1} - d_1 \leq (N-1)F_{\mathrm{conv}}/k$. When many replicas are used, as in the present case where typically $N > 100$, a path that is converged according to this criterion may have a distribution of replicas that is far from being even, as illustrated in Fig. S7.

In order to improve the distribution of replicas along the path, SNEB includes a more global criterion for the distance between replicas obtained from the total length of the path and the number of replicas. An ideal distance from the initial state to each of the replicas is calculated as $D_i^{\mathrm{ideal}} = (i-1)\bar{d}$ where $\bar{d} =$ is the average distance between the replicas in the current path. The parallel spring force is then evaluated with respect to this ideal location of the replica

$$\boldsymbol{F}^{sSNEB}_{i\|} = k(D_i^{\mathrm{ideal}} - D_i)/(2\bar{d})\hat{\boldsymbol{\tau}}_i, \tag{S8}$$

where $D_i = \sum_{j=1}^{i-1} d_j$ is the distance along the path to the current location of the replica. This ensures equal spacing of the replicas upon convergence within the general NEB approach, which includes a spring force and force projections based on a tangent estimate. Distribution of replicas along a path based on D_i^{ideal} was introduced by E and coworkers in the 'string' method[13,14] but there spring forces are not included.

With the two modifications described above, the force acting on an image, i, in an SNEB calculation becomes

$$\boldsymbol{F}^{SNEB}_i = -\nabla V(\boldsymbol{R}_i) + (\nabla V(\boldsymbol{R}_i)\cdot\hat{\boldsymbol{\tau}}_i)\hat{\boldsymbol{\tau}}_i + \boldsymbol{F}^{sSNEB}_{i\|} + \boldsymbol{F}^s_{i\perp}. \tag{S9}$$

The climbing replica scheme to move the replica with highest energy to the saddle point[15] can be used in conjunction with the SNEB method. There, the force acting on the replica with highest energy, i_{cl}, is

$$\boldsymbol{F}_{i_{\mathrm{cl}}} = -\nabla V(\boldsymbol{R}_{i_{\mathrm{cl}}}) + 2(\nabla V(\boldsymbol{R}_{i_{\mathrm{cl}}})\cdot\hat{\boldsymbol{\tau}}_{i_{\mathrm{cl}}})\hat{\boldsymbol{\tau}}_{i_{\mathrm{cl}}}. \tag{S10}$$

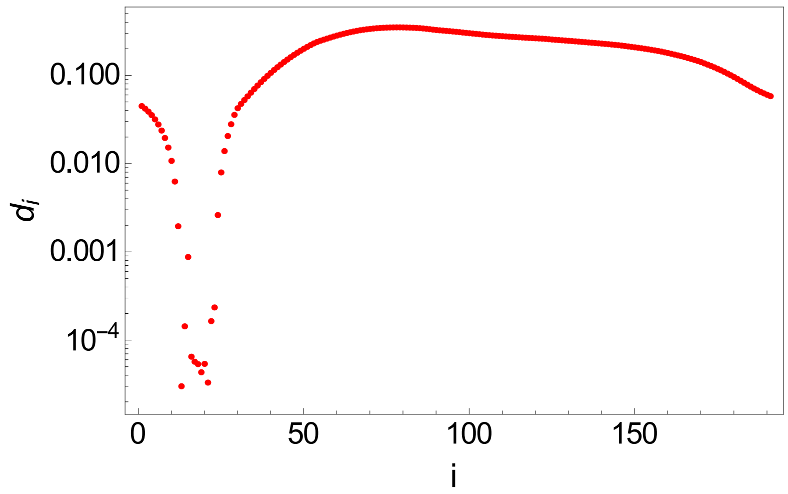

Figure S7. Distance between replicas in a path obtained in a regular NEB calculation for the nucleation of a 60°dislocation in Ge/Si. A total of 192 replicas where included in this segement of the path. The spring force was chosen to be $k = 1$ eV/Å and the convergence tolerance was F_{conv} = 0.02 eV/Å.

When the climbing replica scheme is used with SNEB, the ideal position of a replica, D_i^{ideal}, needs to be modified according to

$$D_i^{\text{ideal}} = \begin{cases} (i-1)\frac{D_{i_{\text{cl}}}}{(i_{\text{cl}}-1)} & \text{if } i < i_{\text{cl}} \\ D_{i_{\text{cl}}} + (i - i_{\text{cl}})\frac{D_N - D_{i_{\text{cl}}}}{N - i_{\text{cl}}} & \text{if } i > i^{\text{cl}}. \end{cases} \tag{S11}$$

The value of $\bar{d}$ in eqn. (S8) is then different for the two parts of the path, before and after the climbing replica.

References

1. Hornstra, J. Dislocations in the diamond lattice. *Journal of Physics and Chemistry of Solids* **5**, 129 – 141 (1958).
2. Bacon, D., Barnett, D. & Scattergood, R. Anisotropic continuum theory of lattice defects. *Progress in Materials Science* **23**, 51 – 262 (1980).
3. Wang, C.-Z., Li, J., Ho, K.-M. & Yip, S. Undissociated screw dislocation in si: Glide or shuffle set? *Applied Physics Letters* **89** (2006).
4. Pizzagalli, L., Godet, J. & Brochard, S. Glissile Dislocations with Transient Cores in Silicon. *Phys. Rev. L* **103**, P065505 (2009).
5. Pizzagalli, L., Godet, J., Guénolé, J. & Brochard, S. Dislocation cores in silicon: new aspects from numerical simulations. *Journal of Physics: Conference Series* **281**, 012002 (2011).
6. Lopatin, S., Pennycook, S. J., Narayan, J. & Duscher, G. Z-contrast imaging of dislocation cores at the gaas/si interface. *Applied Physics Letters* **81**, 2728–2730 (2002).
7. Wang, Y. & Ruterana, P. The strain models of misfit dislocations at cubic semiconductors hetero-interfaces. *Applied Physics Letters* **103** (2013).
8. Mills, G., Jónsson, H. & Schenter, G. K. Reversible work transition state theory: application to dissociative adsorption of hydrogen. *Surf. Sci.* **324**, 305–337 (1995).
9. Jónsson, H., Mills, G. & Jacobsen, K. W. *Nudged Elastic Band Method for Finding Minimum Energy Paths of Transitions, in Classical and Quantum Dynamics in Condensed Phase Simulations* (World Scientific, 1998).
10. Jónsson, H. Simulation of surface processes. *Proceedings of the National Academy of Sciences* **108**, 944–949 (2011).
11. Henkelman, G. & Jónsson, H. Improved tangent estimate in the nudged elastic band method for finding minimum energy paths and saddle points. *J. Chem. Phys.* **113**, 9978–9985 (2000).

12. Maras, E., Trushin, O., Stukowski, A., Ala-Nissila, T. & Jónsson, H. Global transition path search for dislocation formation in ge on si(001). *Comput. Phys. Commun.* **205**, 13 – 21 (2016).

13. E, W., Ren, W. & Vanden-Eijnden, E. String method for the study of rare events. *Phys. Rev. B* **66**, 052301 (2002).

14. E, W., Ren, W. & Vanden-Eijnden, E. Simplified and improved string method for computing the minimum energy paths in barrier-crossing events. *J. Chem. Phys.* **126**, 164103 (2007).

15. Henkelman, G., Uberuaga, B. P. & Jónsson, H. A climbing image nudged elastic band method for finding saddle points and minimum energy paths. *J. Chem. Phys.* **113**, 9901 (2000).
